# Sox9 Gene Transfer Enhanced Regenerative Effect of Bone Marrow Mesenchymal Stem Cells on the Degenerated Intervertebral Disc in a Rabbit Model

**DOI:** 10.1371/journal.pone.0093570

**Published:** 2014-04-01

**Authors:** Wei Sun, Kai Zhang, Guangwang Liu, Wei Ding, Changqing Zhao, Youzhuan Xie, Junjie Yuan, Xiaojiang Sun, Hua Li, Changsheng Liu, Tingting Tang, Jie Zhao

**Affiliations:** 1 Department of Orthopaedic Surgery, Shanghai Ninth People's Hospital, Shanghai Jiao Tong University School of Medicine, Shanghai, PR China; 2 Shanghai Key Laboratory of Orthopaedic Implants, Shanghai, PR China; 3 Department of Orthopaedic Surgery, the Affiliated Hospital of Xuzhou Medical College, Xuzhou, PR China; 4 Department of Orthopaedic Surgery, the Central Hospital of Xuzhou, Affiliated Hospital of Medical College of Southeast University, Xuzhou, PR China; 5 The State Key Laboratory of Bioreactor Engineering, East China University of Science and Technology, Shanghai, PR China; Georgia Regents University, United States of America

## Abstract

**Objective:**

The effect of Sox9 on the differentiation of bone marrow mesenchymal stem cells (BMSCs) to nucleus pulposus (NP)-like (chondrocyte-like) cells in vitro has been demonstrated. The objective of this study is to investigate the efficacy and feasibility of Sox9-transduced BMSCs to repair the degenerated intervertebral disc in a rabbit model.

**Materials and Methods:**

Fifty skeletally mature New Zealand white rabbits were used. In the treatment groups, NP tissue was aspirated from the L2-L3, L3-L4, and L4-L5 discs in accordance with a previously validated rabbit model of intervertebral disc degeneration and then treated with thermogelling chitosan (C/Gp), GFP-transduced autologous BMSCs with C/Gp or Sox9-transduced autologous BMSCs with C/Gp. The role of Sox9 in the chondrogenic differentiation of BMSCs embedded in C/Gp gels in vitro and the repair effect of Sox9-transduced BMSCs on degenerated discs were evaluated by real-time PCR, conventional and quantitative MRI, macroscopic appearance, histology and immunohistochemistry.

**Results:**

Sox9 could induce the chondrogenic differentiation of BMSCs in C/Gp gels and BMSCs could survive in vivo for at least 12 weeks. A higher T2-weighted signal intensity and T2 value, better preserved NP structure and greater amount of extracellular matrix were observed in discs treated with Sox9-transduced BMSCs compared with those without transduction.

**Conclusion:**

Sox9 gene transfer could significantly enhance the repair effect of BMSCs on the degenerated discs.

## Introduction

Degenerative disc disease (DDD) is considered one of the major causes of low back pain [1]. Intervertebral disc degeneration (IVDD) is characterised by a decrease in the function and number of viable cells in the disc, accompanied by a reduction in the synthesis of extracellular matrix (ECM) components such as aggrecan and type II collagen [2]. Current treatments for DDD, such as conservative treatment (medications and physiotherapy) and surgical treatment (spinal fusion or total disc replacement), may alleviate pain but fail to address the underlying problem.

Cell-based tissue engineering, which aims to restore IVD structure and function, represents a promising approach to IVD repair or regeneration. Nucleus pulposus (NP) cell transplantation has been reported to effectively retard IVDD [3]. However, the harvest of NP cells from normal discs could induce degeneration in the donor disc [4], whereas cells derived from degenerative discs show an altered phenotype, increased senescence [5] and decreased expression of matrix components [2]. As NP cells possess a chondrocyte-like phenotype [6], bone marrow mesenchymal stem cells (BMSCs), which are capable of differentiating into a chondrocyte-like phenotype when appropriately stimulated [7], [8], have shown promise as a suitable cell source to be widely used for IVD regeneration [9]–[11].

The pre-modulation of BMSCs prior to implantation has been reported to enhance BMSC therapy [12], [13]. Studies have demonstrated that BMSCs can be differentiated into a chondrocyte-like phenotype by adding transforming growth factor-β (TGF-β) [14] or bone morphogenetic proteins (BMPs) [15], [16] to a pellet culture. However, these methods are unfeasible for tissue engineering strategies. Furthermore, exposure to high concentrations of BMP2 or TGF-β can result in osteogenesis and hypertrophy [17]–[19].

Sox9, a transcription factor belonging to the SOX (Sry-type HMG box) protein family, is essential for chondrogenesis and type II collagen synthesis and has been termed a “master regulator” of the chondrocyte phenotype [20], [21]. Previous studies demonstrated the expression of Sox9 in NP cells [6], and a decrease in its expression has been associated with IVDD [22], suggesting that Sox9 could be a potential therapeutic target. Indeed, Nenadovich et al [23] and Paul et al [24] used an adenoviral vector expressing Sox9 to infect a chondroblastic cell line and IVD cells, which increased Sox9 and type II collagen production in vitro. They also injected Sox9 directly into the IVDs of rabbits and reported that the IVD architecture was preserved for 5 weeks. Despite these encouraging reports, the introduction of exogenous functional cells to replenish the disc cell population may offer more potential for disc repair or regeneration because the success of gene therapy requires a significant number of responding cells, which are limited in degenerated discs. Previous studies have demonstrated that Sox9 gene transfer could promote the chondrogenesis of BMSCs [25], [26], differentiate BMSCs into NP-like cells and improve NP matrix production in vitro [12]. Our recent research has further demonstrated the chondrogenic effect of Sox9 on BMSCs in vitro and its successful application in articular cartilage repair in vivo [27]. However, to date, the application of Sox9-transduced BMSCs in IVD repair has been rarely reported. We hypothesised that Sox9-transduced BMSCs may effectively promote IVD repair or regeneration.

In this study, we aimed to investigate the efficacy and feasibility of Sox9-transduced BMSCs to repair the degenerated IVD in a rabbit model. This method may provide a new strategy for improved IVD repair or regeneration without any requirement for exogenous growth factors.

## Materials and Methods

### Cell harvest and transduction

As we reported previously [27], BMSCs were harvested from 1-year-old New Zealand white rabbits. The marrow was aspirated from iliac, BMSCs were isolated and cultured. The 3rd-passage cells were transduced with adenovirus containing either green fluorescent protein (GFP) (Adv-GFP) or both GFP and Sox9 (Adv-Sox9, Invitrogen). The transduction efficiency was determined by examining GFP-positive BMSCs under a fluorescence microscope (Leica DM 4000B, Germany). Western blot was performed to detect Sox9 expression on the 3rd, 7th, 10th and 14th days after transduction.

### Preparation and characterisation of chitosan-glycerophosphate gels

Sterile chitosan and disodium β-glycerophosphate (supplied by East China University of Science and Technology) were mixed in appropriate proportions to prepare a thermogelling chitosan solution (C/Gp) as previously described [28].

For the microstructural analysis, samples were freeze-dried for 48 h, sprayed with gold for 40 s and then imaged with a scanning electron microscope (SEM) (Hitachi, S4800, Japan).

### Embedding of BMSCs into C/Gp gels

Third-passage cells were trypsinised and counted. An appropriate number of cells were mixed with the C/Gp solution at room temperature to a final concentration of 1×10^6^/ml and prepared for transplantation.

### Analysis of cell viability

For analysis of cell viability, the thermogelling cell suspension was allowed to gel at 37°C in a humidified atmosphere of 5% CO_2_ for 10 min before the addition of culture medium (DMEM, 10% FBS, 1% P/S) to the formed gels. The medium was changed every 3 days. The viability of the encapsulated BMSCs was assessed using a Live/Dead assay kit (Abcam, Sigma-Aldrich). The cell-gel constructs were incubated in a standard working solution at room temperature for 30 min and then observed under a confocal laser scanning microscope (Nikon A1R, Japan). The nuclei of live cells stained bright green, and dead cells stained red. Additionally, cell viability was also assessed by the MTT assay as previously described [29].

### Gene expression analysis using real-time PCR

Total RNA was isolated from monolayer BMSCs and from BMSCs in C/Gp gels using the Trizol reagent (Invitrogen) according to the manufacturer's instructions. After the reverse transcription reaction, real-time PCR was performed with an ABI 7500 system using SYBR Premix Ex Taq™ (Takara, Dalian, China) according to the manufacturer's instructions. The real-time PCR conditions were as follows: 35 cycles of 95°C for 15 s and 60°C for 34 s. A dissociation stage was added to the end of the amplification procedure. No nonspecific amplification was observed as determined by the dissolve curve. GAPDH was used as an internal control. The primer sequences used for this analysis are listed in Table 1.

**Table 1:** Sequence of primers.

Gene	Sequence (5′-3′)	Product size (bp)
Aggrecan	F: TAAACCCGGTGTGAGAACCG	176
	R: CCTGGGTGACAATCCAGTCC	
Collagen I	F: ACATCCCGCCAGTCAC	119
	R: CACGTCATCGCACAAGA	
Collagen II	F: GGATAGACCCCAACCAAGGC	122
	R: GCTGCTCCACCAGTTCTTCT	
Collagen X	F: GAAAACCAGGCTATGGAACC	256
	R: GCTCCTGTAAGTCCCTGTTGTC	
Sox9	F: GGTGCTCAAGGGCTACGACT	273
	R: GGGTGGTCTTTCTTGTGCTG	
GAPDH	F: GGAATCCACTGGCGTCTTCA	122
	R: GGTTCACGCCCATCACAAAC	

### Animal study

This study was carried out in strict accordance with the recommendations in the Guide for the Care and Use of Laboratory Animals of the National Institutes of Health.The protocol was approved by the Animal Care and Experiment Committee of Shanghai Jiaotong University School of Medicine. All surgery was performed under general anesthesia, and all efforts were made to minimize suffering. Fifty healthy skeletally mature New Zealand white rabbits (male, 1-year-old, and 2.5–3 kg) were used. Disc degeneration was induced 2 weeks before the treatment via a validated puncture method [10]. The rabbits were tranquilised by the intramuscular injection of ketamine hydrochloride (40 mg/kg) and xylazine (2.5 mg/kg). NP (5–8 mg wet weight) was aspirated from the L2-L3, L3-L4 and L4-L5 discs using an anterolateral approach with a 21-gauge needle on a 10-ml syringe.

The rabbits were divided into 5 groups according to different treatment: nonsurgical (normal, n = 10), puncture surgery (degeneration, n = 10), puncture surgery followed by C/Gp injection (C/Gp, n = 10), puncture surgery followed by GFP-transduced autologous BMSCs with C/Gp transplantation (GFP, n = 10) and puncture surgery followed by Sox9-transduced autologous BMSCs with C/Gp transplantation (Sox9, n = 10). The C/Gp solution or Sox9/GFP-transduced BMSCs-C/Gp suspension (15 μl) was carefully injected into the discs through a 27-gauge insulin injector. To prevent cell leakage, the needle was slowly pulled out 10 min after injection to allow time for the solution to gel in the NP cavity.

### Magnetic resonance imaging (MRI) analysis

The rabbits were tranquilised as described above and placed in the standard human knee coil in the supine position. A 3.0-T MR scanner (Magnetom Verio; Siemens, Germany) was used to obtain T2-weighted images (repetition time  = 3,800 ms, echo time  = 98 ms, 130×130 mm field of view, slice thickness of 2 mm with a 0-mm gap) and T2 mapping images (repetition time  = 1,000 ms, echo time  = 12.3, 24.6, 36.9, 49.2 and 61.5 ms, 130×130 mm field of view, slice thickness of 2 mm with a 0-mm gap) at 6 and 12 weeks post-treatment. T2-weighted images were used for the conventional evaluation. T2 mapping images were used to calculate the relaxation time (T2) of NP for the quantitative evaluation of IVDD by the segmentation method [30].

### Computed tomography (CT) analysis

In this study, CT scanning was performed to investigate the presence of osteophyte in the IVD region between two flanking vertebrae at 12 weeks post-treatment. A GE Light Speed 16 CT scanner (GE Healthcare, London, UK) was used to obtain coronal and sagittal reconstruction images.

### Macroscopic evaluation

Rabbits were sacrificed by an intravenous overdose of pentobarbital at 6 or 12 weeks post-treatment. Their spines were harvested, and the discs were isolated with both upper and lower vertebral bodies completely attached. The specimens were fixed in 4% paraformaldehyde for 24 h and decalcified in 0.05% EDTA for 4 weeks. The specimens were then cut longitudinally at the centre of the discs for gross morphological observations.

### Histological and immunohistochemical evaluation

After the macroscopic observation, the specimens were embedded in paraffin and cut into 5 μm sections. These sections were stained with haematoxylin and eosin and with safranin O for evaluation. The degenerative changes were graded by 2 blinded observers using the histological grading scale proposed by Masuda et al [31] (Table 2).

**Table 2:** Histological grading scale by Masuda et al [31].

**I. Annulus fibrosus**
**Grade**
1. Normal pattern of fibrocartilage lamellae (U-shaped in the posterior aspect and slightly convex in
the anterior aspect) without ruptured fibres and without a serpentine appearance anywhere within the
annulus
2. Ruptured or serpentined patterned fibres in less than 30% of the annulus
3. Ruptured or serpentined patterned fibres in more than 30% of the annulus
**II. Border between the annulus fibrosus and nucleus pulposus**
**Grade**
1. Normal
2. Minimally interrupted
3. Moderate/severe interruption
**III. Cellularity at the nucleus pulposus**
**Grade**
1. Normal cellularity with large vacuoles in the gelatinous matrix
2. Slight decrease in the number of cells and fewer vacuoles
3. Moderate/severe decrease (>50%) in the number of cells and vacuoles
**IV. Matrix of the nucleus pulposus**
**Grade**
1. Normal gelatinous appearance
2. Slight condensation of the extracellular matrix
3. Moderate/severe condensation of the extracellular matrix

Histological grading scale based on four categories of degenerative changes. A normal disc is defined as 4 points. A higher score implies more severe degeneration in nucleus pulposus and annulus fibrosus.

Type II collagen expression was detected using a mouse monoclonal antibody (1∶200; Abcam, Cambridge, MA) and a horseradish peroxidase-conjugated anti-mouse antibody (1∶50; Dako, Denmark), followed by colour development with diaminobenzidine tetrahydrochloride (DAB, Dako). The results of type II collagen staining were quantified in mean density with the IPP version 6.0 software (Media Cybernetics, Bethesda, MD, USA).

### Evaluation for survival of BMSCs

Some discs from the GFP and Sox9 groups were frozen in liquid nitrogen immediately after harvest and cryosectioned axially at 8 μm. For double staining, the sections were incubated with an anti-GFP monoclonal antibody (1∶500; Abcam) and an anti-Sox9 polyclonal antibody (1∶200; Abcam). Alexa 488 and 555 (1∶200; Molecular Probes) were used as the fluorescent second antibodies. Finally, the sections were counterstained with 4′, 6-diamidino-2-phenylindole (DAPI, Invitrogen) to stain the nuclei and then observed under a fluorescent microscope. To compare the relative cell density between the two groups, cells in five sections crossing the center of each disc, with three discs per group, were counted. Actually, the calculation of the cells in NP was performed using the ×200 magnification images with counting (DAPI+GFP)-positive/all DAPI-positive cells in the GFP group, while counting (DAPI+GFP+Sox9)-positive/all DAPI-positive cells in the Sox9 group.

### Statistical analysis

The statistical analysis was performed with SPSS 16.0 (Chicago, USA). Student's t-test, repeated measure ANOVA and Fisher's LSD post hoc test were used to analyse the data. *P*<0.05 was considered significant.

## Results

### Chondrogenic differentiation of BMSCs in monolayer

**Figure 1:**
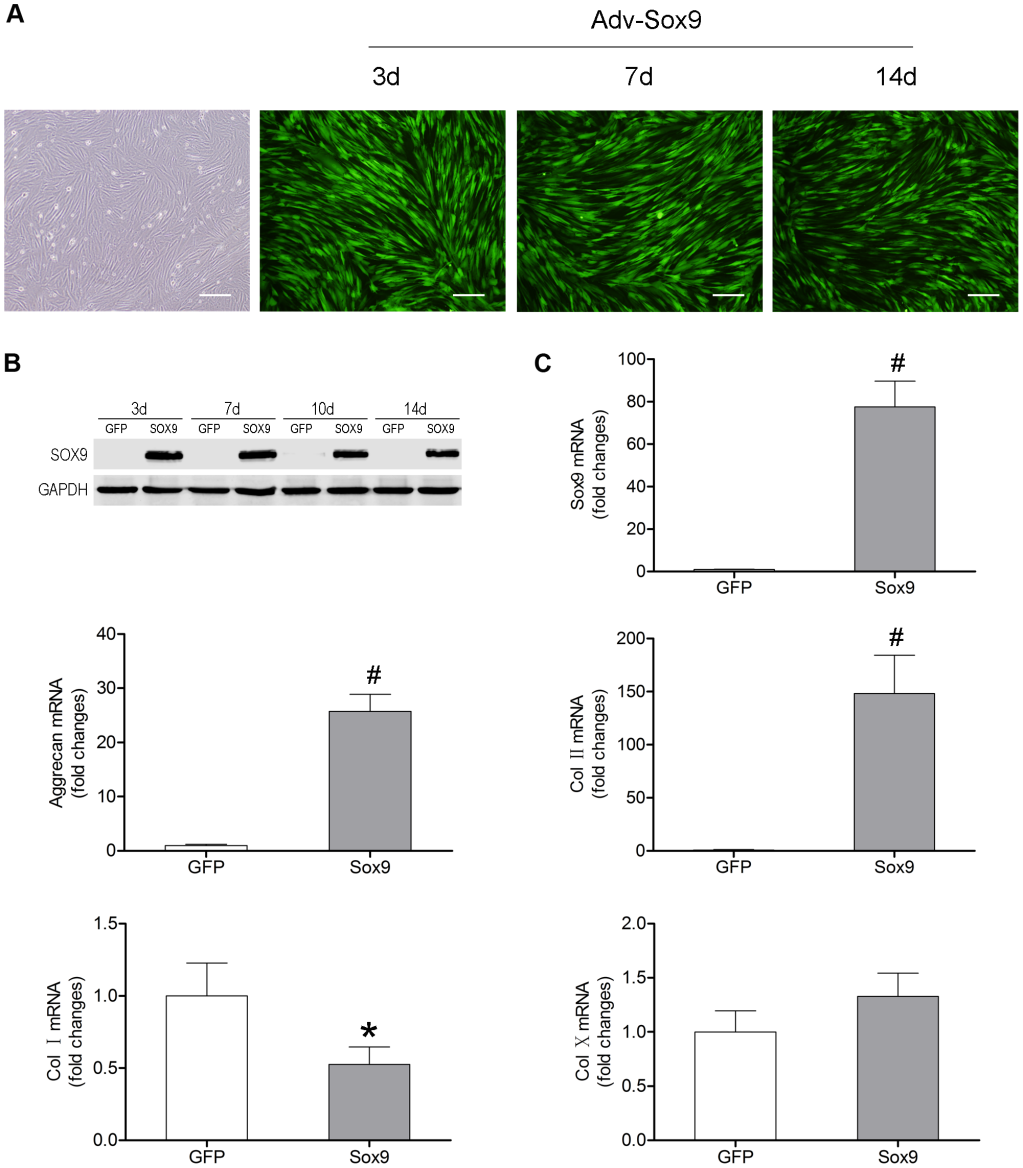
Sox9-transduced BMSCs in monolayer. (A) P3 BMSCs exhibited a uniform fibroblast-like shape, and fluorescence microscopy showed Sox9 transduction efficiency at days 3, 7 and 14. (B) Sox9 expression was evaluated by western blot at days 3, 7, 10 and 14 after transduction. (C) Real-time PCR for the expressions of *Sox9*, *Aggrecan*, *Col II*, *Col I* and *Col X* was performed at day 21 after transduction using *GAPDH* as a housekeeping gene. All results represent the mean±SD of triplicate samples. *P<0.05, #*P*<0.01. Scale bar = 200 μm.

The transduction efficiency and the Sox9 expression demonstrated the successful Sox9 transduction of BMSCs. The mean±SD percentages of GFP-positive cells were 92.7±2.1%, 86.7±4.1% and 78.3±7.6% at 3, 7 and 14 days after transduction, respectively (Fig. 1A). Sox9-transduced BMSCs expressed the highest level of Sox9 at day 3. Although it gradually decreased over time, the expression of Sox9 was higher in Sox9-transduced BMSCs than in GFP-transduced BMSCs at all time points (Fig. 1B).

Real-time PCR was performed for the evaluation of BMSCs differentiation. The results showed that the expressions of *Col II*, *Aggrecan* and *Sox9* were up-regulated 148-fold (*P*<0.01), 26-fold (*P*<0.01) and 78-fold (*P*<0.01) at day 21 after Sox9 transduction, respectively, and that the expression of *Col I* was down-regulated by 47% (*P*<0.05) in Sox9-transduced BMSCs compared with GFP-transduced BMSCs. However, overexpression of Sox9 had no significant effect on *Col X* expression (*P*>0.05) (Fig. 1C). These data indicate that Sox9 transduction can differentiate BMSCs in monolayer into a chondrocyte-like phenotype, and does not induce hypertrophy.

### Chondrogenic differentiation of BMSCs in C/Gp gels

**Figure 2:**
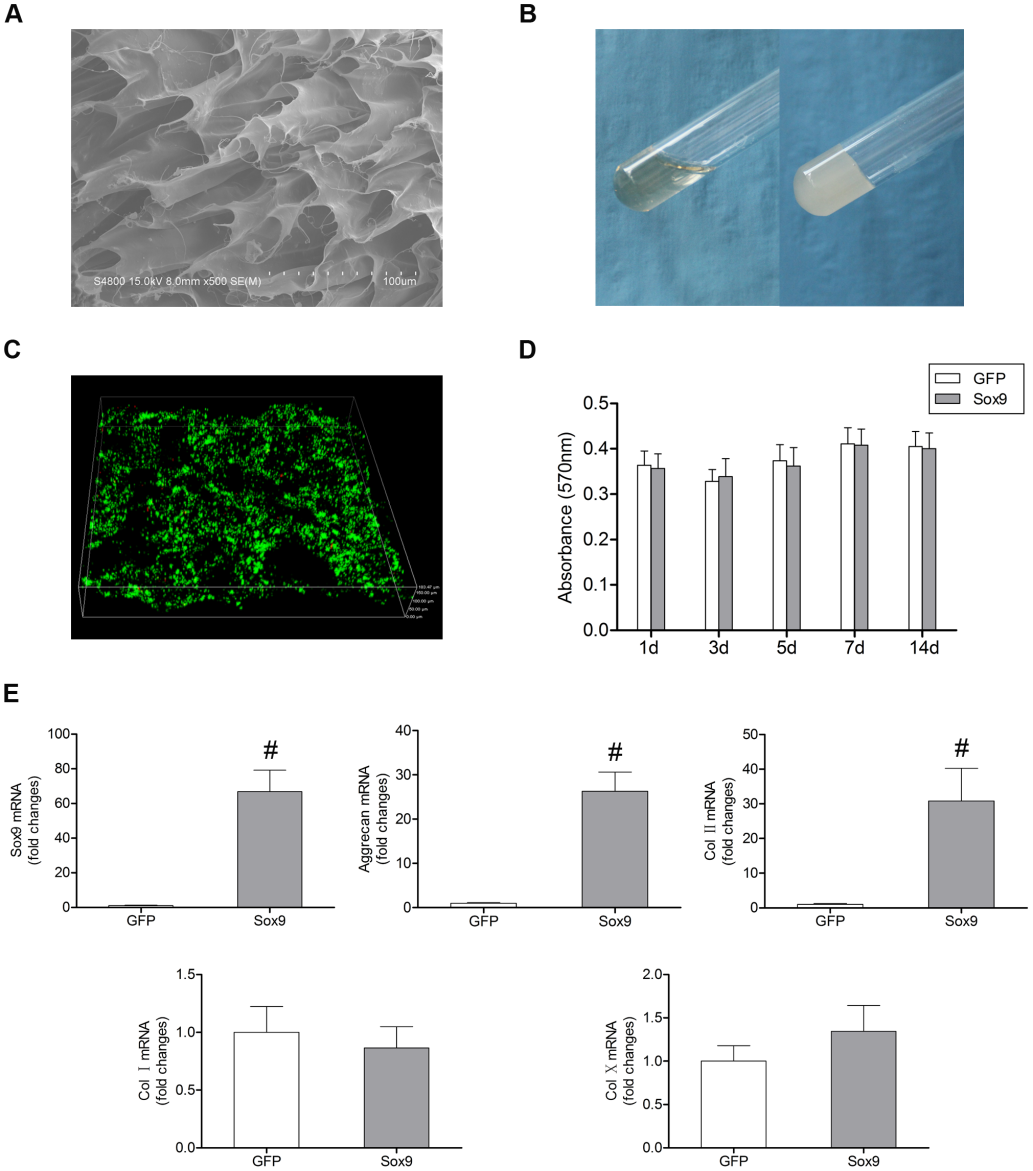
Sox9-transduced BMSCs in C/Gp gels. (A) Scanning electron microscopy observation of the C/Gp gels. (B) The C/Gp was liquid at room temperature and gelled at 37°C. (C) Live/dead assay of the BMSCs encapsulated in C/Gp gels after 3 days of culture. (D) The MTT assay was used to assess the BMSC proliferation in C/Gp gels over 14 days. (E) The gene expression analysis of the BMSCs in C/Gp gels after 21 days of culture. #*P*<0.01. Scale bar = 25 μm.

A typical microstructure of the C/Gp gels is shown in Fig. 2A. The C/Gp gels showed highly interconnected and homogeneously distributed pores, with a regular pore diameter in the range of 30–50 μm. Furthermore, the C/Gp solution was observed to be liquid at room temperature and gel at 37°C (Fig. 2B). To evaluate the biocompatibility of the C/Gp gels, the analysis of cell viability was performed. The live/dead cell staining showed a relatively homogeneous distribution of BMSCs within C/Gp gels and that most of the BMSCs were viable after 3 days of culture (Fig. 2C). The MTT assay showed that proliferation proceeded equally in both groups with no significant difference during 14 days of culture (Fig. 2D). These suggest that the C/Gp gels possess satisfactory biocompatibility.

The chondrogenic differentiation of BMSCs in C/Gp gels is demonstrated in Fig. 2E. The expression levels of *Col II*, *Aggrecan* and *Sox9* in Sox9-transduced BMSCs encapsulated in C/Gp gels were significantly higher than in GFP-transduced BMSCs at day 21 after transduction (*P*<0.01), whereas the expression of *Col I* was lower but not significantly different (*P*>0.05). Overexpression of Sox9 did not significantly upregulate the expression of *Col X* (*P*>0.05). The findings demonstrate that Sox9 transduction can induce chondrogenic differentiation of BMSCs in C/Gp gels with no significant hypertrophy.

### MRI and CT findings

**Figure 3:**
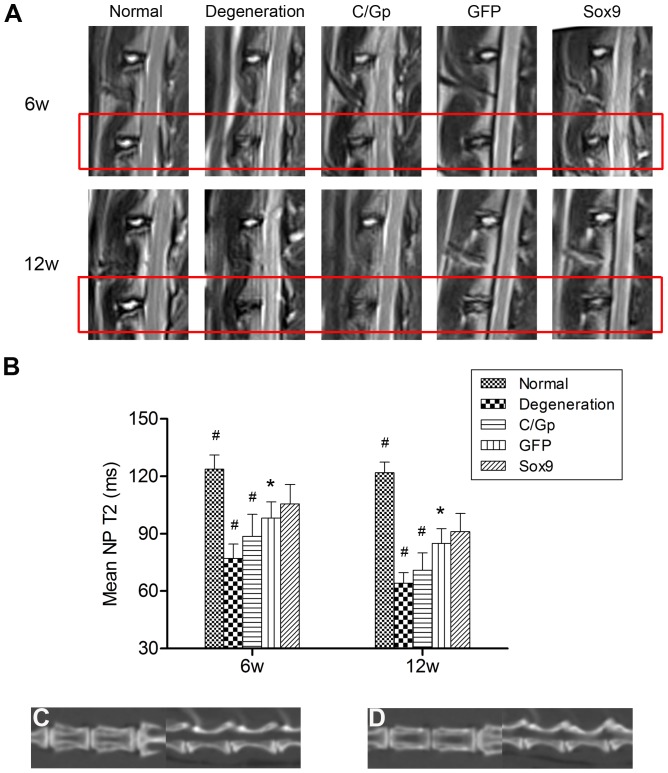
Imaging studies. (A) T2-weighted images of the treated discs (outlined by the red box) and adjacent control discs at 6 and 12 weeks post-transplantation. (B) The mean T2 of NP at 6 and 12 weeks post-transplantation. *P<0.05, #P<0.01 vs. the Sox9 group. Coronal and sagittal CT reconstruction images for the GFP (C) and Sox9 (D) groups.

The T2-weighted MR images for discs are shown in Fig. 3A. Significant decreases in the NP area and signal intensity were observed in the degeneration and C/Gp groups throughout the study period. In contrast, the Sox9 and GFP groups showed fewer degenerative changes over the same period. The Sox9 group showed less decrease in the NP area and T2-weighted signal intensity compared with that in the GFP group. The mean T2 of the NP in the Sox9 group was significantly higher than those of the degeneration, C/Gp and GFP groups at both time points (Fig. 3B). The coronal and sagittal CT reconstruction demonstrated no obvious osteophyte formation in the GFP and Sox9 groups (Fig. 3C and D).

### Macroscopic findings

**Figure 4:**
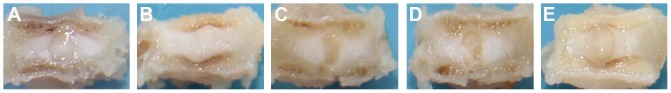
Macroscopic views of the representative discs at 12 weeks post-transplantation: : (A) normal control group; (B) degeneration group; (C) C/Gp group; (D) GFP group and (E) Sox9 group.

The gross appearance of representative discs at 12 weeks post-treatment is shown in Fig. 4A-E. The normal discs showed a well-defined translucent NP. The depletion of NP, connective tissue invasion and an unidentified border between annulus fibrosus (AF) and NP were observed in the degeneration group. The C/Gp group showed a loss of NP structure and significant infolding of the AF. In contrast, although less translucent, the NP of the Sox9 and GFP groups showed relatively well preserved structure. The NP of the Sox9 group more closely resembled the normal NP than that of the GFP group.

### Histological and immunohistochemical fingdings

**Figure 5:**
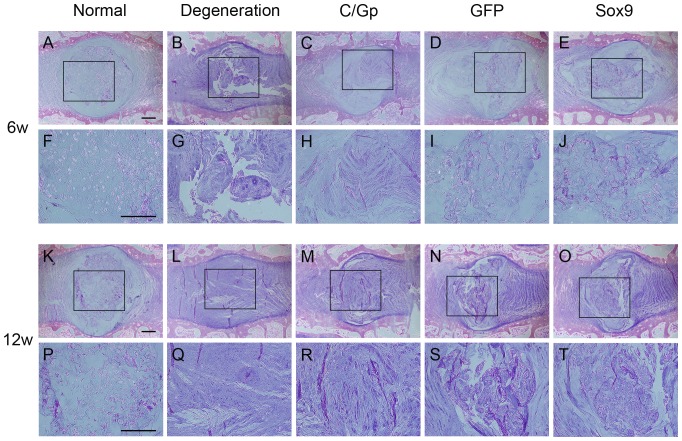
Haematoxylin and eosin staining of sections from the 5 groups at 6 (A–J) and 12 weeks (K–T) post-transplantation. Scale bar = 500 μm.

**Figure 6:**
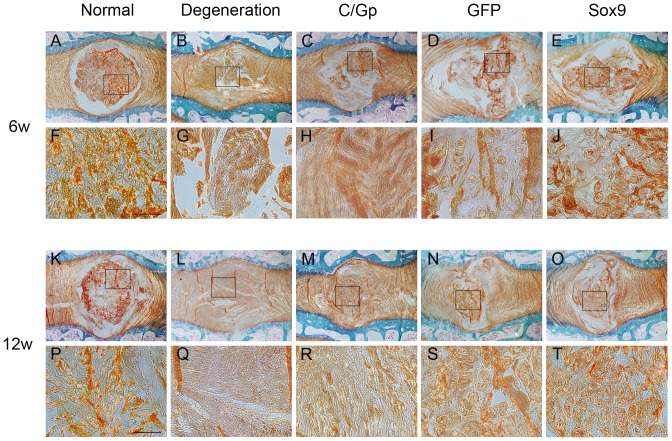
Safranin O staining of sections from the 5 groups at 6 (A–J) and 12 weeks (K–T) post-transplantation. Scale bar = 200 μm.

At 6 weeks post-treatment, the degeneration group exhibited a disorganised structure of the NP and a serpentine appearance of the AF. Although the border between the AF and NP could be identified, an apparently decreased number of chondrocyte-like cells were observed in the C/Gp group. In contrast, the Sox9 and GFP groups showed a relatively well-preserved NP structure, an identifiable border between the AF and NP and a number of chondrocyte-like cells in the NP region (Fig. 5A–J). Safranin O staining in the Sox9 and GFP groups showed clusters of cells with a slight condensation of proteoglycan-rich ECM; the discs of the Sox9 group were more similar to those of the normal group, whereas the degeneration and C/Gp groups showed a relatively faint NP staining (Fig. 6A–J), suggesting the decrease in proteoglycan.

At 12 weeks post-treatment, in the degeneration group, the NP disappeared with the apparent invasion of connective tissue. The C/Gp group showed an unidentified border between the AF and NP and a further decrease in the number of chondrocyte-like cells in the NP region, which was occupied by disorganised fibrocartilaginous tissue. In contrast, despite the condensation of ECM and infolding of the AF, the Sox9 and GFP groups preserved a partial NP structure and arrested the decrease in cellularity. Compared with the GFP group, the Sox9 group preserved more organised NP structures and less condensation of the ECM as well as a more distinct border between the AF and NP (Fig. 5K–T). Safranin O staining showed relatively dark NP staining in the Sox9 and GFP groups. The Sox9 group exhibited a relatively abundant proteoglycan compared with the GFP group. The degeneration and C/Gp groups showed a reddish ECM, indicating fibrosis (Fig. 6K–T).

**Figure 7:**
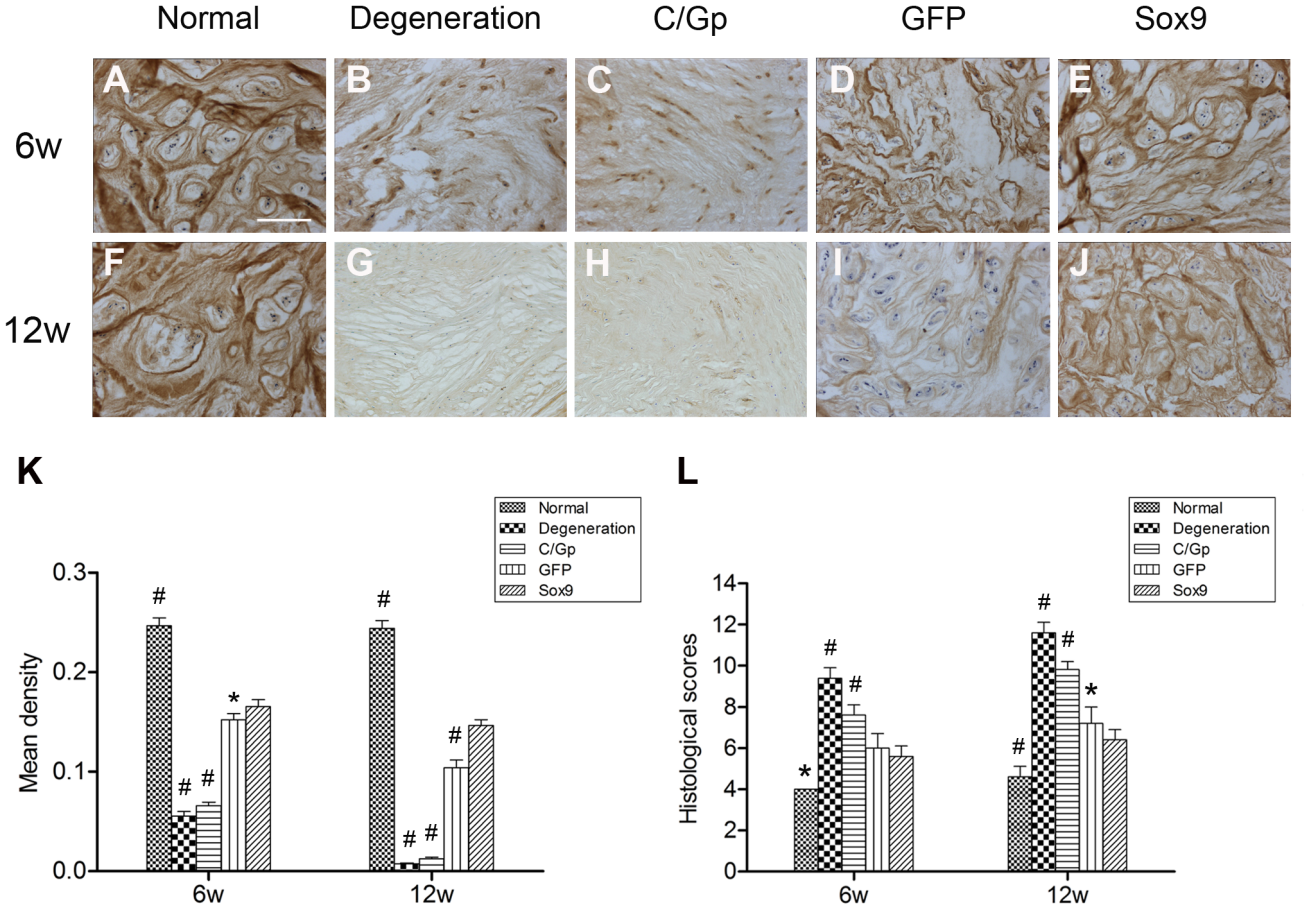
Type II collagen immunohistological staining and histological scores. Representative sections from the 5 groups at 6 (A–E) and 12 weeks (F–J) post-transplantation. (K) Quantitative analysis of the type II collagen staining using mean density. (L) Histological scores of discs from the 5 groups at 6 and 12 weeks post-treatment. *P<0.05, #*P*<0.01 vs. the Sox9 group. Scale bar = 200 μm.

Immunohistochemical staining showed little or no type II collagen in the NP region in the degeneration and C/Gp groups but did demonstrate the presence of type II collagen in the other groups at 12 weeks post-treatment. The staining was significantly stronger in the Sox9 group than in the GFP group at both time points (Fig. 7A–K).

According to the histological grading scale, the average scores in the Sox9 group (5.6±0.5 and 6.4±0.5 at 6 and 12 weeks post-treatment, respectively) were significantly lower than the scores in the degeneration group (9.4±0.5 and 11.6±0.5, respectively), the C/Gp group (7.6±0.5 and 9.8±0.4, respectively) and the GFP group 6.0±0.7 and 7.2±0.8, respectively) (Fig. 7L).

### Survival of BMSCs at 12 weeks post-transplantation

**Figure 8:**
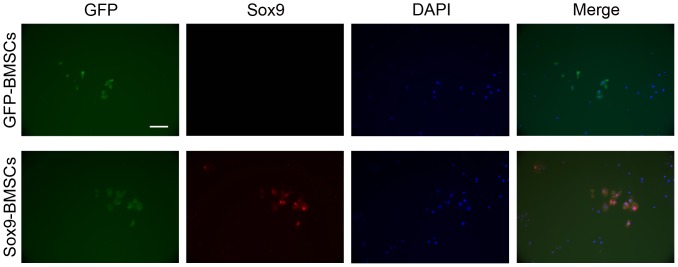
Double staining of the transplanted BMSCs using antibodies against GFP (green) and Sox9 (red) at 12 weeks post-transplantation. DAPI was used to stain the cell nucleus (blue), and the colours are labelled in the image. Scale bar = 200 μm.

No GFP-positive cells were detected in the normal, degeneration or C/Gp groups, whereas some GFP-positive cells were observed in the central region of the NP in the Sox9 and GFP groups at 12 weeks post-transplantation, demonstrating the survival of the transplanted BMSCs. Some GFP-positive BMSCs in the Sox9 group costained together with Sox9 (Fig. 8), indicating that the Sox9-transduced BMSCs expressed Sox9 successfully in vivo. Additionally, no statistically significant difference in cell density between the two groups was noticed (*P*>0.05).

## Discussion

This study investigated the efficacy of Sox9-transduced BMSCs for IVD repair in a well-established rabbit disc degeneration model. We demonstrated that Sox9-transduced BMSCs could survive for up to 12 weeks after transplantation and effectively retard progressive degeneration through improved ECM production.

To allow for cell delivery, proliferation, differentiation and matrix synthesis and therefore assist tissue regeneration, a scaffold appears to be essential [12], [32]. The thermogelling C/Gp can be injected into the NP in liquid form, enabling the use of a minimally invasive approach. Once inside the body, the cell-C/Gp suspension will gel in the NP. Recently, Vadala et al [33] reported that intradiscal injection of BMSCs leaded to osteophyte formation due to cell leakage. One of the main reasons is that BMSCs were injected in saline rather than a viscoelastic cell delivery scaffold. More importantly, they used a model of IDD induced by annular stab. Such discs have a high swelling pressure and resist the injection of any volume of substance. In this study, we used the gelatinous scaffold to help cell retention in the discs of a NP aspiration model of IDD with a significant reduction of the intradiscal swelling pressure, alleviating cell leakage and the associated osteophyte formation (Fig. 3C and D). To evaluate the biocompatibility of the C/Gp gels, we used the stereological method to investigate the viability of BMSCs in three-dimensional (3D) C/Gp gels, which helped further mimic and study the in vivo status of the BMSCs in IVD, in contrast to most previous studies that only investigated the viability of cells on the surface of hydrogels [29], [34]. Our results demonstrated that BMSCs maintained high viability and proliferation after 14 days of culture (Fig. 2C and D), suggesting that the C/Gp gels possess satisfactory biocompatibility to allow for cell survival and proliferation, which is consistent with previous studies [28], [35].

In line with previous studies [12], [27], we demonstrated that the overexpression of Sox9 could differentiate monolayer BMSCs into a chondrocyte-like phenotype (Fig. 1C). For Sox9-transduced BMSCs to be further applicable to a tissue engineering strategy, ensuring that the chondrocyte-like phenotype was maintained in the C/Gp gels was important. In the present study, we demonstrated that compared with the GFP-transduced BMSCs, the Sox9-transduced BMSCs showed significantly high expression levels of *Sox9*, *Col II* and *Aggrecan* when cultured in 3D C/Gp gels (Fig. 2E). This finding suggests that the C/Gp gels can maintain the chondrogenic effect of Sox9 on BMSCs, which agrees well with the previous study [12]. Furthermore, we also demonstrated that overexpression of Sox9 had no significant effect on *Col X* expression, which indicates that hypertrophy is not induced during the in vitro chondrogenic differentiation of BMSCs by overexpression of Sox9 (Fig. 1C and 2E).

To further test the feasibility of our hypothesis, an in vivo study designed to use Sox9-transduced BMSCs to repair degenerated discs was performed.

In addition to the conventional T2-weighted signal intensity, T2 mapping—a biochemical MRI technique to calculate T2—was applied to offer a quantitative assessment of the degenerative changes. Unlike T2-weighted signal intensity, T2 is neither scanner- nor image parameter-dependent [36]. Rather, it reflects an intrinsic property of the tissue, providing information about water content, collagen orientation and matrix structure [37]. The T2 of IVD has been demonstrated to correlate well with the water and proteoglycan contents [36]. Our results revealed that discs treated with Sox9-transduced BMSCs showed a significantly high signal intensity (Fig. 3A) and T2 (Fig. 3B) of the NP, representing relatively high water and proteoglycan contents. This observation was supported by its more hydrated gross appearance (Fig. 4E) and more intensive safranin O staining (Fig. 6E, J, O and T). These findings indicate that Sox9-transduced BMSCs can promote degenerated disc repair with proteoglycan restoration and thus increased hydration within the IVDs.

Histological evaluation revealed that discs in the Sox9 group exhibited a relatively well-preserved NP structure showing a chondrocyte-like morphologic appearance compared with those of the other groups (Fig. 5E, J, O and T). We showed in the current study that discs treated with Sox9-transduced BMSCs showed a relatively uniform distribution of ECM that stained strongly positive for safranin O (Fig. 6E, J, O and T) and type II collagen immunoreactivity (Fig. 7E and J), suggesting the improved production of aggrecan and type II collagen by the Sox9-transduced BMSCs. This may support at the protein level previous studies that revealed that Sox9 was essential for certain cartilage matrix genes such as Col II and Aggrecan [38], [39]. Our results further identified the important role of Sox9 in stimulating the production of ECM, which may help restore the biochemical properties and maintain the histological features of the NP. Better repair was also supported by the lower degeneration grade for the Sox9 group (Fig. 7L), although the transplantation of GFP-transduced BMSCs was effective to some degree.

Sobajima et al [40] and Zhang et al [41] showed that BMSCs could survive for up to 24 weeks after transplantation in the IVDs of rabbits. In our study, we demonstrated that BMSCs could survive in vivo for at least 12 weeks. Some GFP-positive BMSCs detected in the NP region in the Sox9 group expressed the Sox9 protein (Fig. 8), suggesting the success of gene therapy and its responsibility for the observed improvement in all parameters compared with the GFP group. These findings further support the notion that the successful stem cell therapy of IVDD may require the aid of supplementary gene transfer, scaffolds and other techniques to overcome the complexities of IVDD and the limitations of stem cells [42], [43].

There are several limitations of the present study. First, this injury model may not represent IVDD in humans. Second, although several potential NP markers were reported [44], we still cannot identify whether or not the Sox9-transduced BMSCs differentiated to authentic NP cells due to the lack of NP-specific marker without interspecies variation. Nevertheless, our outcomes indeed demonstrated that Sox9-transduced BMSCs could effectively retard progressive degeneration through improved ECM production. Further studies are needed to elucidate NP-specific markers to help decide which phenotype should be targeted in future stem cell-based therapy for IDD. Third, if growth or transcription factors could be integrated into scaffolds and released in a controlled manner over time, then the risks related to a viral vector could be avoided. Finally, to achieve better efficacy, the deficient nutrition and altered biomechanics in the degenerated discs must be addressed, and the annulus fibrosus and cartilage endplate should also be taken into account in future studies.

In conclusion, the results of this study demonstrated that Sox9 gene transfer could enhance the repair effect of BMSCs on degenerated discs through improved ECM production. Our study provided evidence for developing a new strategy for the promotion of IVD repair or regeneration without adding any other exogenous growth factors. Further studies will be necessary to evaluate the long-term efficacy of this therapy.
